# Use of a hemostatic matrix for control of bleeding during flexible endoscopic septotomy for Zenker’s diverticulum

**DOI:** 10.1055/a-2794-7551

**Published:** 2026-02-09

**Authors:** Camille Boustani, Simone Dibitetto, Roberto De Sire, Antonio Capogreco, Roberta Maselli, Cesare Hassan, Alessandro Repici

**Affiliations:** 1 71541 MedStar Georgetown University Hospital, Washington D. C., United States; 2 9268 Endoscopy Unit, IRCCS Humanitas Research Hospital, Milan, Italy; 3 437807 Department of Biomedical Sciences, Humanitas University, Pieve Emanuele, Milan, Italy

Adverse events during flexible endoscopic septotomy (FES) are rare, but include bleeding, perforation, and infection 1 2 . We present a case ( Video 1 ) of intraprocedural significant bleeding during FES for Zenker’s diverticulum (ZD), controlled with a self-assembling peptide gel. The transparent viscous gel used is a hemostatic material in the form of a prefilled syringe. It is intended for bleeding prophylaxis after endoscopic mucosal resection or endoscopic submucosal dissection (ESD), as well as hemostasis of both intra-procedural and post-procedural bleeding 3 4 . Previous randomized controlled trials have shown that the use of this hemostatic matrix reduced the need for thermal therapy during ESD for intraprocedural bleeding 5 .

The video depicts a case of intraprocedural significant bleeding during flexible endoscopic septotomy (FES) for Zenker’s diverticulum (ZD), controlled with a self-assembling peptide gel.Video 1

The use of non-diathermic intervention was indicated due to the inadequate visualization of the bleeding vessel as a result of the significant hemorrhage, and the failure of traditional diathermic coagulation techniques. This self-assembling peptide gel was utilized to help reduce the risk of perforation associated with applying diathermic current to this area. The use of this hemostatic matrix to control severe intra-procedural bleeding during FES for a ZD has not been previously described.

Intraprocedural bleeding during FES for a ZD is a rare, though known adverse event of this procedure 1 2 . We present a non-diathermic strategy utilizing a self-assembling peptide gel to successfully control a significant intraprocedural bleeding during FES ( Video 1 ). This has the advantage of limiting thermal injury to deeper structures and maintaining adequate visualization of tissue planes.

Endoscopy_UCTN_Code_TTT_1AO_2AD
